# Toxic Activity, Molecular Modeling and Docking Simulations of *Bacillus thuringiensis* Cry11 Toxin Variants Obtained via DNA Shuffling

**DOI:** 10.3389/fmicb.2018.02461

**Published:** 2018-10-17

**Authors:** Alvaro Mauricio Florez, Miguel Orlando Suarez-Barrera, Gloria M. Morales, Karen Viviana Rivera, Sergio Orduz, Rodrigo Ochoa, Diego Guerra, Carlos Muskus

**Affiliations:** ^1^RG Microbial Ecology: Metabolism, Genomics & Evolution, Microbiomas Foundation, Chía, Colombia; ^2^Laboratorio de Biología Molecular y Biotecnología, Universidad de Santander, Bucaramanga, Colombia; ^3^Escuela de Medicina, Facultad de Salud, Universidad Industrial de Santander, Bucaramanga, Colombia; ^4^Grupo Biologa Funcional, Laboratorio de Prospección y Diseo de Biomoléculas, Escuela de Biociencias, Universidad Nacional, Sede Medellín, Colombia; ^5^Programa de Estudio y Control de Enfermedades Tropicales PECET, Unidad de Biologa Molecular y Computacional-UBMC, Universidad de Antioquía, Medellín, Colombia

**Keywords:** *Bacillus thuringiensis*, Cry11, DNA shuffling, docking, *Aedes aegypti*, *Culex quinquefasciatus*

## Abstract

The Cry11 family belongs to a large group of δ-endotoxins that share three distinct structural domains. Among the dipteran-active toxins referred to as three-domain Cry11 toxins, the Cry11Aa protein from *Bacillus thuringiensis* subsp. *israelensis* (*Bti*) has been the most extensively studied. Despite the potential of *Bti* as an effective biological control agent, the understanding of Cry11 toxins remains incomplete. In this study, five Cry11 variants obtained via DNA shuffling displayed toxic activity against *Aedes aegypti* and *Culex quinquefasciatus*. Three of these Cry11 variants (8, 23, and 79) were characterized via 3D modeling and analysis of docking with ALP1. The relevant mutations in these variants, such as deletions, insertions and point mutations, are discussed in relation to their structural domains, toxic activities and toxin-receptor interactions. Importantly, deletion of the N-terminal segment in domain I was not associated with any change in toxic activity, and domain III exhibited higher sequence variability than domains I and II. Variant 8 exhibited up to 3.78- and 6.09-fold higher toxicity to *A. aegypti* than Cry11Bb and Cry11Aa, respectively. Importantly, variant 79 showed an α-helix conformation at the C-terminus and formed crystals retaining toxic activity. These findings indicate that five Cry11 variants were preferentially reassembled from the *cry11Aa* gene during DNA shuffling. The mutations described in loop 2 and loop 3 of domain II provide valuable information regarding the activity of Cry11 toxins against *A. aegypti* and *C. quinquefasciatus* larvae and reveal new insights into the application of directed evolution strategies to study the genetic variability of specific domains in *cry11* family genes.

## Introduction

**FIGURE 1:**
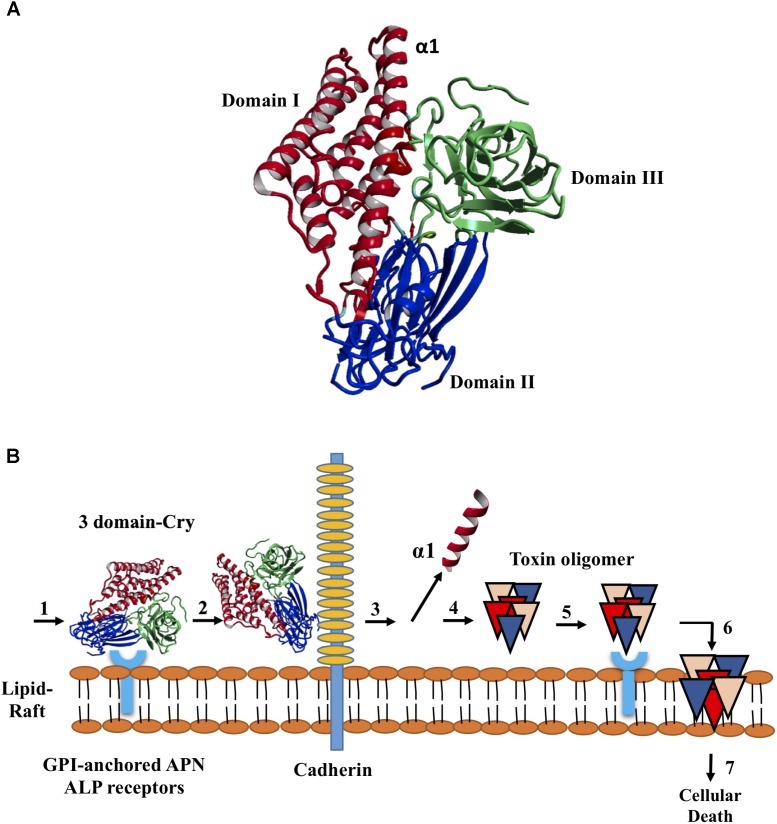
Structure of the Cry toxins, domains, and their mode of action. **(A)** Ribbon diagram of Cry deduced 3D structure. Three domains are colored in red blue and green, respectively. **(B)** Sequential binding mechanism. 1. The toxin binds to GPI-anchored APN and ALP receptors in the lipid rafts; 2. Binding to cadherin receptor 3. Proteolytic cleavage of the helix α1 at *N*-terminal end; 4. *N*-terminal cleavage induces the formation of pre-pore oligomer 5. Increasing of the oligomer binding affinity to GPI-anchored APN and ALP receptors; 6. Oligomer inserts into the membrane, leading to pore-formation and cell lysis; and 7. Cellular death.

*Bacillus thuringiensis* (*Bt*), a Gram-positive bacterium characterized by the production of Cry δ-endotoxins capable of killing insects, has been used since the late 1930s as a biological control agent (Melo et al., 2016). A total of 308 holotype toxins are clustered into 75 Cry proteins (Crickmore et al., 2014) (revised February, 2018). The tertiary structures of nine Cry toxins determined via X-ray crystallography to date contain three conserved domains with specific functions and implicated in the structural stability of the protein. The domain I is a bundle of 7–8 α helices involved in pore formation, domain II is a β-prism with exposed loops regions involved in receptor binding and, domain III is a β-sandwich and has influence on receptor binding, ion channel formation and insect specificity (Li et al., 1991; Grochulski et al., 1995; Derbyshire et al., 2001; Galitsky et al., 2001; Morse et al., 2001; Guo et al., 2009; Hui et al., 2012; **Figure 1A**).

The mechanisms by which *Bt* induces death in insects are controversial and have not been completely elucidated (Vachon et al., 2012). Currently, there are two mechanisms related to *Bt*-induced toxicity in insects that have been accepted; the sequential binding and signaling pathways (Zhang Q. et al., 2017). The sequential binding mechanism has been extensively studied and is based on the formation of pores in epithelial cells in the midgut of targeted insects, which results in toxin-receptor interactions, osmotic imbalance and cell death (Bravo et al., 2007; Pardo-Lopez et al., 2013). After crystal ingestion, Cry toxins become solubilized, and specific proteases present in the lumen of the midgut activate the toxins, which then bind to specific receptors located in the insect midgut. In some Cry toxins, this event induces the proteolytic removal of helix α1 (Aronson, 2000), triggering Cry toxin oligomerization, insertion of oligomeric structures altering membrane stability, receptor and production of channels or pores, ultimately leading to cell lysis and insect death (Bravo et al., 2004; **Figure 1B**). The signaling pathways is a recent proposed mechanism in which the activation of signaling cascades, leads to increased cyclic AMP and protein kinase activities, resulting in cell death (Zhang et al., 2006). Both of these mechanisms contain gaps. In the sequential binding mechanism, the presence of several types of resistance and the link between proteolysis and pore formation are not understood (Melo et al., 2016). In the signaling pathway mechanism, how Cry toxin-receptor interactions mediate toxic activity is unclear (Melo et al., 2016). In this context, it has been suggested that Cry toxins can cause death based on their ability to induce both pore formation and ion channel activation (Zhang Q. et al., 2017).

The pBtoxis megaplasmid from *Bt* subsp. *israelensis* (*Bti*) contains four Cry proteins encoded by the *cry4Aa*, *cry4Ba*, *cry10Aa*, and *cry11Aa* genes and two Cyt proteins encoded by the *cyt1Aa* and *cyt2Ba* genes (Berry et al., 2002). The *cry* genes produce 134, 128, 78, and 72 kDa polypeptides, respectively, all of which possess larvicidal activity higher than of the Cyt’s proteins (Ben-Dov, 2014). However, the high toxic activity of *Bti* is the result of synergistic interactions between all of them (Ben-Dov, 2014). That is the case of Cyt1Aa that despite the low toxicity, it is highly synergistic with *Bti* Cry toxins and aids to overcome resistance in mosquitoes to Cry toxins (Wirth et al., 1997). Due to the synergistic interactions of *Bt* subsp. *israelensis* toxins, this bacterium has been used worldwide to control mosquito larvae of the genera *Aedes*, *Culex* and *Anopheles*, which are involved in the transmission of diseases including malaria, hemorrhagic fever, dengue fever, lymphatic filariasis, yellow fever (Ben-Dov, 2014), Chikungunya and Zika (Chouin-Carneiro et al., 2016; Gardner et al., 2016; Tsetsarkin et al., 2016).

Cry11Aa from *Bti* is a 72 kDa protoxin that is activated by gut enzymes via the proteolytic removal of 28 residues from its N-terminus and proteolytic cleavage into two fragments of 38 and 30 kDa that remain associated and retain toxicity (Dai and Gill, 1993; Revina et al., 2004; de Barros Moreira Beltrao and Silva-Filha, 2007). The Cry11Aa toxin has higher activity against *Aedes* and *Culex* than against *Anopheles* (Revina et al., 2004; Otieno-Ayayo et al., 2008). In *A. aegypti*, this toxin interacts with two midgut brush border membrane receptors; a GPI anchored and alkaline phosphatase (ALP1) (Fernandez et al., 2006) and also binds to Cyt1Aa as a kind of membrane-bound receptor of Cry11Aa increasing the toxic activity (Perez et al., 2005). Other midgut proteins different to the receptor alkaline phosphatase (ALP1) such as ATP binding protein, increases the toxicity of Cry11Aa against *C. quinquefasciatus* (Zhang L. et al., 2017). Other two toxins, Cry11Bb (94 kDa) and Cry11Ba (81 kDa), share a similar insect specificity and are phylogenetically related to Cry11Aa. Cry11Bb and Cry11Ba are produced by *Bt* subsp. *medellin* and *Bt* subsp. *jegathesan*, respectively (Delecluse et al., 1995; Orduz et al., 1998).

Although the tertiary structure of Cry11 toxins have not been determined by X-ray crystallography, Cry11Aa have been the most studied among this group using protein engineering tools. Several mutations haven been introduced into different domains that are implicated in its toxicity. Therefore, studies have focused on domain I developing N-terminally truncated forms of Cry11Aa (Pang et al., 1992) or modifications in domains II and III altering the interactions with its receptor in the midgut, confirming the importance of these domains for Cry11Aa-mediated toxicity (Fernandez et al., 2005, 2009). Based on phage display and site-directed mutagenesis, the exposed regions of loop α8, β4 and loop 3 in domain II of Cry11Aa have been shown to be involved in the interaction of Cry11Aa with *A. aegypti* brush border membrane vesicles (BBMVs). Specifically, two mutations in loop α8, V262E and E266A, reduced the toxic activity of Cry11Aa against *A. aegypti* (Fernandez et al., 2005). There are also mutations in loop α-8 that are involved in Cry11Aa–ALP1 receptor interaction, that affect the Cyt1Aa and Cry11Aa interaction reducing the synergism between these proteins and decreasing their toxic activity (Perez et al., 2005). Other binding sites have been described for the interaction of Cry11Aa with the receptor ALP1 as an important secondary receptor for Cry11Aa and Cry11Ba (Chen et al., 2017). The involved regions are located in loop 2 of domain II and β18-β19 of domain III of Cry11Aa, which interact with ALP1 regions R^59^–G^102^ and N^257^–I^296^, respectively (Fernandez et al., 2009).

Since the three Cry11 toxins are phylogenetically related and exhibit similar specificity to insect species, it is possible to infer similarities at structural level that can be analyzed after mixing their genes in order to create novel proteins with improved properties. Therefore, considering the lack of studies focused on this approach, we designed a DNA shuffling strategy to obtain variants with increased toxicity to *A. aegypti* and *C. quinquefasciatus.* DNA shuffling has been used alone or in combination with phage display and the staggered extension process via homologous recombination to increase the activity of Cry toxins against specific insect pests (Lucena et al., 2014). This technique is a powerful approach based on recombination between parental genes in a single DNA shuffling reaction following random fragmentation (Stemmer, 1994).

Here, we report five variants that were reassembled from the *cry11Aa* gene that exhibit from moderate to high toxic activities against *A. aegypti* and *C. quinquefasciatus* mosquito larvae. Variant 8 was the most toxic to the mosquito larvae, and variants 23 and 79 displayed important differences in 3D structure, toxin-ALP1 interactions and toxicity in which are implicated domains II and III preferably.

## Materials and Methods

### Microbial Strains, Clone Selection and Gene Constructs

For DNA manipulation, *Escherichia coli* DH5α, JM109 (Promega) and DH5αTOP10(Life Technologies) cells were grown at 37°C in Luria Bertani (LB) culture medium supplemented with ampicillin (50 μg/ml) and X-gal (50 mg/ml). A *Bt* 4Q2-81 strain carrying the pBTM3 plasmid and expressing the *cry11Bb* gene from *Bt* subsp. *medellin* (Restrepo et al., 1997) and a second *Bt* 4Q2-81 strain carrying the pJEG90.1 plasmid and expressing the *cry11Ba* gene from *Bt* subsp. *jegathesan* (Delecluse et al., 1995) were cultured as previously described (Restrepo et al., 1997) in M1 medium supplemented with 30 μg/ml tetracycline and 20 μg/ml erythromycin. Crystal production was evaluated via phase contrast microscopy. *E. coli* DH5α cells harboring the pSV2 plasmid, which carried the *p19* gene upstream of *cry11Aa* from *Bt subsp. israelensis*, were cultured in M1 medium supplemented with 20 μg/ml chloramphenicol; this strain was obtained from Dr. Neil Crickmore from the University of Sussex. The pTOAa, pTOBa-1, pTOBa-2, and pTOBb plasmids carrying the *cry11Aa*, *cry11Ba-1, cry11Ba-2*, and *cry11Bb* genes, respectively, were amplified via PCR and cloned using the TOPO TA Cloning^®^ system (Life Technologies) (**Supplementary Table 1**). To obtain pGEBb-1, the pTOBb vector was digested with *EcoR*I and *BamH*I, releasing an insert of 3.5 kb that was ligated into pGEM7zf (+) (Promega). The DNA shuffling library was cloned into the TA TOPO cloning system. Selected variants were subcloned into the pSV2 expression vector using *Hind*III and *Sac*I and transformed into BMB171 cells in LB supplemented with 6 μg/ml chloramphenicol. The acrystaliferous strain BMB171 was used to produce the variants and was donated by Dr. Ziniu Yu from the State Key Laboratory of Agricultural Microbiology, Huazhong Agriculture University, Wuhan, Hubei, China.

### Isolation of *cry11* Genes via PCR

The *cry11* genes were amplified via PCR using specific primers and plasmid DNA from constructs pBTM3, pSV2 and pJEG90.1 as templates. Briefly, reactions were conducted in a final volume of 50 μl that contained 20 ng of plasmid DNA, 0.5 μM primers, 1× *Taq* polymerase buffer, 0.4 mM dNTPs, 1.5 mM MgCl_2_ and 0.5 U Go*Taq* polymerase (Promega). The amplification conditions were denaturation at 94°C for 5 min followed by 35 cycles of 45 s at 94°C, 45 s at 55°C, and 4 min 30 s at 72°C, and a final extension at 72°C for 10 min. The PCR products were separated via electrophoresis and purified using the PCR Clean-Up System (Promega). The *cry11Ba* gene was obtained via two independent PCRs, producing a 1.8-kb product (denoted Ba1) corresponding to *cry11Ba* with a deletion of 246-bp downstream of the ATG start site and a 0.9-kb fragment (denoted as Ba-2) that contained a 0.75-kb fragment of *cry11Ba* including the stop codon and a 249-bp segment homologous to the multiple cloning site (MCS) of the pHT315 shuttle vector.

### *cry11* Gene Cloning, Insert Validation, and Sequencing

The *cry11* genes obtained via PCR were cloned using the TOPO TA system. Recombinant variants for each product were selected, and plasmid DNA was extracted using the Wizard Plus Minipreps kit (Promega). Verification of each insert was performed via digestion of 50 ng of DNA with *EcoR*I, followed by separation via agarose gel electrophoresis. The released inserts were also used as templates for PCR to confirm the presence of the *cry11* genes in the inserts. Each reaction was conducted in a final volume of 25 μl that contained 0.4 μM each of the forward primer 5′-TTAGAAGATACGCCAGATCAAGC-3′ and the reverse primer 5′-CATTTGTACTTGAAGTTGTAATCCC-3′ (Bravo, 1997; Bravo et al., 1998) in 1× *Taq* polymerase buffer, 0.4 mM dNTPs, 2.5 mM MgCl_2_ and 0.12 U Go*Taq* polymerase. The amplification conditions were 5 min at 94°C followed by 35 cycles of 45 s at 94°C, 45 s at 51°C, and 1 min at 72°C and a final extension step of 6 min at 72°C. pTOAa containing a 2.5-kb insert, pTOBa-1 containing a 1.6-kb insert, pTOBa-2 containing a 0.78-kb insert and pTOBb containing a 3.5-kb insert were sequenced by Macrogen, Inc. (Seoul, South Korea) using M13/T7 primers and the primer pair pCR4F (5′-GATAACAATTTCACACAGGA-3′) and pCR4R (5′-TTGTAAAACGACGGCCAGTG-3′).

### Test Primers for Reassembly via PCR

The pTOAa, PTOBa-1 and pTOBa-2 constructs were used as templates in 50-μl PCRs that contained 0.32 μM PCR4 primers as described in **Supplementary Table 1**, 1× *Pfx* polymerase buffer, 0.4 mM dNTPs, 2.5 mM MgCl_2_ and 0.5 U *Pfx50* polymerase (Life Technologies) in 1× reaction buffer that contained 1 mM MgSO_4_. The amplification conditions were denaturation at 94°C for 4 min followed by 35 cycles of 45 s at 94°C, 45 s at 59–67°C, and 4 min 30 s at 68°C and a final extension step of 10 min at 68°C. The PCR products were separated via electrophoresis and purified using the Wizard purification system (Promega).

To generate the pGE7 construct, 50 ng of pGEBb-1 plasmid DNA was used as a template. The PCR was performed in a final volume of 50 μl that contained 0.32 μM of each primer as described in **Supplementary Table 1**, 1× *Pfx* polymerase buffer, 0.3 mM dNTPs, 1 mM MgSO_4_ and 0.5 U *Pfx50* polymerase. The PCR conditions were denaturation for 4 min at 94°C followed by 35 cycles of 94°C for 45 s, 68°C for 45 s, and 68°C for 4 min 30 s and a final extension step at 68°C for 10 min. The PCR products were separated via agarose gel electrophoresis and purified using the Wizard^®^ SV Gel and PCR Clean-Up System (Promega).

### DNA Shuffling

Three micrograms of each PCR product obtained from the TA cloning constructs and from pGEBb with lengths of 2.5 kb (*cry11Aa*), 1.6 kb (*cry11Ba*-1), 0.78 kb (*cry11Ba*-2), and 3.5 kb (*cry11Bb*) were mixed in 25 μl of 50 mM Tris–HCl, pH 7.4, and 10 mM of MnCl_2_. In the same tube, 0.0006 U DNase I (Life Technologies) was added to a final volume of 50 μl. The reaction was incubated between 5 and 20 min at room temperature to optimize production of fragments ranging between 25 and 250 bp. The reaction was stopped by adding 25 μl of 25 mM EDTA. The DNase I digestion products were separated via electrophoresis and purified with the QIAEX II Gel Extraction Kit (QIAGEN). Forty microliters of the pooled of purified fragments were used as template for a PCR without primers in 1× *Pfx* buffer, 0.3 mM dNTPs, and 2.5 U *Pfx50* polymerase in a final volume of 50 μl under the following conditions: 94°C for 3 min, 45 cycles of 94°C for 30 s, 48°C for 3 min and 68°C for 1 min (with a 12-s increase in extension time per cycle), and a final extension step at 68°C for 7 min. The products for reassembly were validated via agarose gel electrophoresis and purified using the Wizard PCR Clean-Up system.

The full-length sequences were amplified using two sets of primers, PCR4F/R and PGE7F/R, and the combination of these primers, PCR4F/PGE7R, along with a template of 1 μl of the products of the primerless PCR, to a final volume of 50 μl. The PCR was conducted in 1× *Pfx*50 buffer, 0.3 μM dNTPs, 0.3 μM primers, and 5 U *Pfx50* under the following conditions: 4 min at 94°C, 25 cycles of 94°C for 45 s, 55°C for 1 min, and 68°C for 4 min (with a 20-s increase in extension time per cycle), and a final extension step at 68°C for 10 min. The second PCR product, corresponding to the DNA shuffling product, was analyzed via agarose gel electrophoresis and purified using the Wizard PCR Prep DNA Purification System.

### Cloning, DNA Sequencing and Homology Analysis

The shuffled PCR products were cloned using the TOPO Zero Blunt PCR cloning kit (Life Technologies) according to the manufacturer’s instructions and chemically competent *E. coli* DH5αTOP10 cells (Life Technologies). Sequencing data were used to select clones according to their open reading frame (ORF) and DNA identity to the parental genes. Plasmid DNA from each clone was isolated using a Wizard Minipreps kit, and the DNA was sent to Macrogen, Inc., South Korea, for sequencing. The forward and reverse primers used for sequencing were as follows: M13 forward (-20): 5′-GTAAACGACGGCCAG-3′; M13 reverse, 5′-CAGGAAACAGCTATGAC-3′. Gene homology analysis was performed using BLASTn and BLASTx, available at^1^. Sequence alignments were performed using ClustalW (Thompson et al., 1994), available on the web^2^.

### Transformation of *Bacillus thuringiensis*

The PCR products were cloned using TOPO Zero Blunt PCR cloning kit and then subcloned into the pSV2 expression vector using *Hind*III and *Sac*I. The resulting vectors were transformed into BMB171 cells via electroporation using a Bio-Rad Micropulser^TM^. BMB171 cells were grown in LB-glycine 0.12% up to an OD600 of 0.15, corresponding to the early exponential phase, and then transformed with 500 ng of the constructs. The electroporation conditions were 2 kV/cm, 200 Ω, and 25 μF for 4 ms. Transformed cells were revitalized via incubation in 500 μl of LB for 2 h at 30°C at 50 rpm. Two hundred microliters of transformed cells were plated on 60-mm Petri dishes containing LB agar supplemented with 6 μg/ml chloramphenicol. Colony counts and percent efficiency of transformation were calculated after 48 h. Endospore and crystal formation was evaluated via scanning electron microscopy (SEM).

### Cultures, Solubilization, Cry Protein Quantification, and SDS–PAGE

Cultures expressing variants 1, 8, 23, 79, and 81 and recombinant Cry11Aa and Cry11Bb, including the *Bt* acrystaliferous BMB171 strain, were grown in 10 ml of LB supplemented with chloramphenicol (6 μg/ml) for 7 days at 30°C and 300 rpm. After 48 h of incubation, the culture purity was confirmed via microscopic observation of spores, crystals, and lysed cells. The final culture was collected via centrifugation at 11,200 × *g* for 15 min at 4°C. The supernatant was discarded, and 1 ml of 1 M NaCl was added to the pellet, which was shaken for 1 h at 30°C and 50 rpm to neutralize protease activity. Then, the suspension was washed twice with 1 ml of 1× PBS and centrifuged at 11,200 × *g* for 5 min at 4°C. The number of spores was determined via heat shock using 100 μl of each culture and incubated at 72°C for 20 min. Afterward, the samples were incubated at 4°C for 10 min and were diluted by 10^-1^ to 10^-5^ in a final volume of 100 μl. The dilutions were plated on LB agar supplemented with chloramphenicol (6 μg/ml) and incubated at 30°C for 24 h.

To quantify Cry protein production, 200 μl of the final sporulated culture of each variant was solubilized by adding 800 μl of solubilization buffer (50 mM NaOH, 10 mM EDTA, pH 11.7), incubating the culture at 4°C overnight and centrifuging the culture at 25,200 × *g* for 1 h at 4°C. The supernatant was collected, and the volume was adjusted to 1 ml with Tris-base (0.1 M, pH 7.4). The protein concentration was determined using the Bradford protein assay (Bradford, 1976) and was confirmed via SDS–PAGE using bovine serum albumin (BSA) as a standard. The protein samples were electrophoresed on 10% SDS–PAGE gels at 80 V for 90 min using a Bio-Rad mini protein system (Bio-Rad Laboratories). A total of 5 μg of protein was loaded per lane, and the protein bands were visualized by staining with Coomassie Brilliant Blue R-250 solution for 30 min.

### SEM

The final cultures were centrifuged at 11,200 × *g* at 4°C for 10 min, and the precipitate was washed twice in 1× PBS. The pellet was resuspended in 1/10 of the original volume, and 100 μl of the samples were placed on glass slides and dried overnight at room temperature. The samples were coated with a thin layer of gold on a Denton Desk Vacuum IV and analyzed using a JEOL JSM 5010 LV scanning electron microscope.

### Half Lethal Concentration (LC_50_) in *A. aegypti* and *C. quinquefasciatus* Larvae

Each bioassay consisted of two replicates, each with 30 first instar larvae in 1 ml at 24°C for each variant at 7 different concentrations under the same environmental conditions. A total of 420 larvae were used for each variant. Larval mortality was determined by counting the number of live larvae after 24 and 48 h, and 50% lethal concentrations were determined statistically via Probit analysis which employ a transformation from sigmoid dose-response curve to a straight linear and then analyzed by a regression on the relationship. The calculation of the average lethal concentration (LC_50_) was made using the R-Project Software^3^.

### 3D Structure Prediction and Validation and Secondary Structure Analysis of Non-conserved Regions

The amino acid sequences of parental Cry11Aa and variants 8, 23, and 79 were modeled via threading methodology using the free local server I-TASSER^4^. From the five models obtained by the program, the first model of each structure was selected according to the best C and TM scores. These structures were geometrically and energetically validated to assess the quality of the generated 3D model using different servers, such as the Ramachandran SWISS-MODEL^5^, the Z-score and energy graph in the ProSA-web server^6^, ERRAT^7^, and Verify3D^8^. The structures obtained for variants 8, 23, and 79 were aligned with the structure of Cry11Aa. For variant 79, complementary analysis based on the predicted secondary structure of non-conserved regions was performed using JPred^9^. Subsequently, the *ab initio* method was used to predict the 3D structure of variant 79 using Robetta server^10^.

### Molecular Docking of Cry11 Domains With ALP1

Parental Cry11Aa and variants 8, 23, and 79 were analyzed to identify their interactions with the receptor ALP1 from *A. aegypti* (UniProtKB ID; Q16WV8). For this protein, two regions that interact with Cry11Aa were identified by epitope mapping. These regions are located within R59-G102 and N257-I296 in ALP1, which interact with residues in loop α8 of Cry11Aa domain II and residues R561-N570 in Cry11Aa domain III, respectively (Fernandez et al., 2009). The structure of ALP1 was modeled using the I-TASSER server taking into account folding recognition by threading. The model was evaluated according to Z-scores obtained using ProSA-web (see text footnote^6^), and a Ramachandran plot was generated using Swiss-MODEL (see text footnote^5^). For docking analysis, the interactions between Cry11Aa and ALP1 regions were analyzed using the Cry11Aa-interacting domains as peptides with rigid conformations based on the predicted 3D structures obtained in the previous step. The ALP1 structure and the peptides obtained from Cry11Aa and each variant were parameterized using AutoDockTools^11^ via the addition of polar hydrogens to each residue’s side chain to facilitate the formation of hydrogen bonds. The structures were also treated with Gasteiger partial charges to facilitate electrostatic interactions among other molecular entities. Docking analysis was performed using AutoDock Vina^12^ considering an exhaustiveness set to 80, which is proportional to the length of the ligand. For the simulated interactions with both regions of ALP1, 3D grid cubic boxes with sides of 32 Å in length and a grid space of 1.0Å were located on the defined active site center, covering all the residues of interest and allowing the entrance of the full peptide structures into the protein cavities. Subsequently, the different docking conformations for each variant were illustrated and analyzed using LIGPLOT^13^.

## Results

### Parental *cry11* Genes

*cry11Aa*, *cry11Ba*, and *cry11Bb* were used as parental genes to be fragmented in DNA shuffling based on their closely phylogenetical relationship, similarities at structural protein level and toxic specificity to similar insect species. This approach has been used for *in vitro* recombination of families of homologous genes in order to create novel proteins with improved properties and is useful for those in which the three-dimensional structure is unknown. In contrast to other random mutagenesis protocols, this technique introduces mutations by random DNA fragmentation and PCR reassembly in a cyclic process that alternates gene diversification, screening and selection of functional variants (Stemmer, 1994).

Four PCR products of 3.5, 2.7, 1.8, and 0.9 kb corresponding to *cry11* genes were obtained (**Supplementary Figure 1A**), as confirmed via DNA sequencing. The 3.5-kb PCR product contained a 2.2-kb fragment encoding the Cry11Bb protein. Three segments were also identified downstream of the last stop codon. The first segment consisted of 234-bp and showed 93% identity to *cry11Bb2* (accession number HM68615.1). The second segment was a 129-bp fragment that showed 93% identity to the complementary strand of the *IS2140* insertion element (accession number M23740.1) and was used to distinguish the reassembled products from the *cry11Bb* gene. The third segment, a 173-bp fragment, showed 83% identity to the complementary strand of the *cry30Aa* gene (accession number AJ251978.1).

The 2.7-kb PCR product contained a 1.9-kb fragment encoding the Cry11Aa protein as well as two additional segments of 580- and 90-bp that were identified upstream of the first ATG and downstream from the stop codon, respectively. The 580-bp fragment was homologous to the p19 accessory protein gene (GenBank: CAD30080.1) and was used to distinguish the reassembled products from *cry11Aa* gene. The downstream 90-bp segment was homologous to the MCS of pSV2. Sequence analysis showed that the 1.8-kb and 0.9-kb PCR products from *cry11Ba*, denoted as Ba1 and Ba2, respectively, shared a 407-bp segment. A deletion of 246-bp downstream of ATG start site was used to recognized the reassembled products from *cry11Ba* gene. No mutations were detected in the DNA sequences of the PCR products of any of the parental genes.

### Assembly of Full-Length *cry11* Genes

The primers used for DNA shuffling were tested via conventional PCR and random fragmentation, and the results indicated that the parental gene amplifications were successful (**Supplementary Figure 1A**). The purified PCR products (**Supplementary Figure 1B**) were mixed and treated with DNase I for 7, 8, or 9 min. However, only the products treated for 8 min (**Supplementary Figure 1C**) generated fragments between 25 and 200-bp. These products were reassembled, resulting fragments between 1 and 10-kb (**Supplementary Figure 1D**). After the final assembly using the PCR4F and pGE7R primers, we observed fragments between 0.25 and 2-kb (**Supplementary Figure 1E**). The assembly reaction products were cloned as described in the Materials and Methods. A total of 94 variants were obtained, and 10 of these variants did not contain an insert. For the remaining variants, 34 were <1.0-kb, 14 were between 1.1 and 2-kb, 22 were >2.1-kb and, and 14 did not show homology.

### Characteristics and Sequence Homology of *cry11* Variants

According to the sequence analysis of the Cry11 variants, 14 of them did not show homology to any known endogenous Cry11 toxin. Among those 14 variants, six variants were >2.1-kb and eight were between 1 and 2-kb. The 22 variants that displayed sequence homology and a similar size to the full-length parental genes (>2.1-kb) were clustered into three groups. The first group consisted of variants 1, 8, 23, 28, 54, 79, and 81; these variants contained the p19 gene located upstream from the ATG start site and showed homology to *cry11Aa*. A second group of ten variants, including 16, 36, 51, 57, 61, 68, 71, 75, 77, and 85, showed DNA homology to *cry11Aa* but lacked the p19 gene. The third group of variants, 14, 17, 67, 76, and 86, showed homology to the *cry11B* genes.

Among the 22 variants that were between 1 and 2-kb, 14 showed homology to the *cry11B* genes, and eight did not have homology to any of the *cry11* genes used. All 34 variants that were <1-kb showed homology to the *cry11B* genes, and their reassembled products contained only domain III.

Among all variants obtained via DNA shuffling, variants 1, 8, 23, 28, 54, 79, and 81 were selected for characterization. Homology analysis of the deduced amino acid sequences of Cry11Aa and variants 1, 8, 23 and 81 showed a high degree of conservation with few amino acid changes, which were preferentially located in domain III (**Supplementary Figure 2A**). Variants 28 and 54 exhibited 100% identity to Cry11Aa. Therefore, these mutants were excluded from further analysis. Comparative analysis of Cry11Aa, variant 23 (aa 1-643), variant 8 (aa 1-568), and variant 79 (aa 1-551) showed that the two first variants are highly conserved in the extension of the sequences. However, many variations relative to Cry11Aa were present in variant 79 at the end of the sequence, particularly beginning from aa 286. In addition to polymorphisms, several insertions/deletions were present at the end of this sequence (**Supplementary Figures 2B,C**).

Finally, secondary structure analysis of the deduced amino acid sequence from variants 8, 23, and 79 predicted the presence of α helix, β-sheets and loops (**Supplementary Figure 2C**). The accession numbers and particular characteristics of the variants 1, 8, 23, 79, and 81 genes are described in **Table 1**.

**Table 1:** Molecular characteristics of Cry11 variants obtained via DNA shuffling.

				DI	DII	DIII		
						
Variants	GenBank accession number	Identity (%) *cry11Aa*	Mutation rate (%)	Del. (nt)	Subs. (nt)	Ins. (nt)	Subs. (nt)	Del. (nt)
Variant 1	MH068786	84,6	15	219	0	73	6	1
Variant 8	MH068787	87,7	13	219	6	0	13	0
Variant 23	MH068788	98,9	1	9	6	5	2	0
Variant 79	MH068789	80,1	20	326	7	42	21	0
Variant 81	MH068790	90,7	8	153	0	8	2	0
TOTAL				926	19	128	44	1


*D, domain; Del, deletions; Subs, substitutions; Ins, insertions.*

### Protein Expression and Crystal Formation

SDS–PAGE revealed that all variants contained a similar pattern of solubilized proteins, and degradation was not observed (**Supplementary Figure 3A**). According to SEM analysis, variants 1, 8, 23, 79, and 81 as well as the parental proteins Cry11Aa and Cry11Bb form crystals (**Supplementary Figure 3B**). Strain BMB171, which was used as the plasmid recipient for protein expression, did not show crystal formation (**Supplementary Figure 3B**).

### The Selected Variants Exhibited Moderate to High Toxic Activity Against *A. aegypti* and *C. quinquefasciatus.*

**FIGURE 2:**
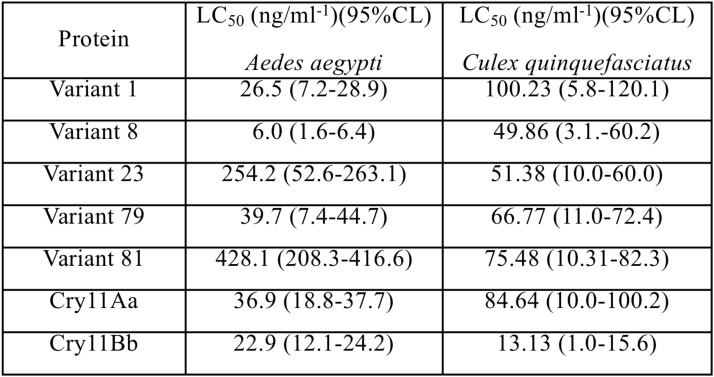
Half lethal concentrations of Cry11 variants obtained via DNA shuffling in *Aedes aegypti* and *Culex quinquefasciatus* larvae. The values are expressed as ng/ml of spore-crystal mixtures, 95% confidence limit (CL).

In accordance with sequence identity, the variants 8, 23, and 79 were reassembled products from the *cry11Aa* parental gene. The toxic activity against *A. aegypti* larvae was up to 3.78- and 6.09-fold higher for variant 8 than for Cry11Bb and Cry11Aa, respectively. No significant differences were observed against *C. quinquefasciatus*. Variants 23 and 79 showed lower and higher mutation rates than Cry11Aa, respectively, although both variants retained toxic activity against *C. quinquefasciatus*. Additionally, variants 23 and 79 exhibited moderate and high toxicity to *A. aegypti*, respectively. Surprisingly, variant 79, despite of high variations still retaining toxic activity. Recombinant Cry11Bb exhibited high toxic activity to both *A. aegypti* and *C. quinquefasciatus* larvae, while variants 23 and 81 exhibited lower toxic activity than control against *A. aegypti*. The results of toxicity assays of five variants for the two types of larvae are shown in **Figure 2**. Based on their mutations and bioassay results, variants 8, 23, and 79 were chosen for analysis of their 3D structure and interaction with ALP1.

### Variants 8 and 23 Are Similar to Their Parental Protein, Whereas Variant 79 Shows Structural Differences

**FIGURE 3:**
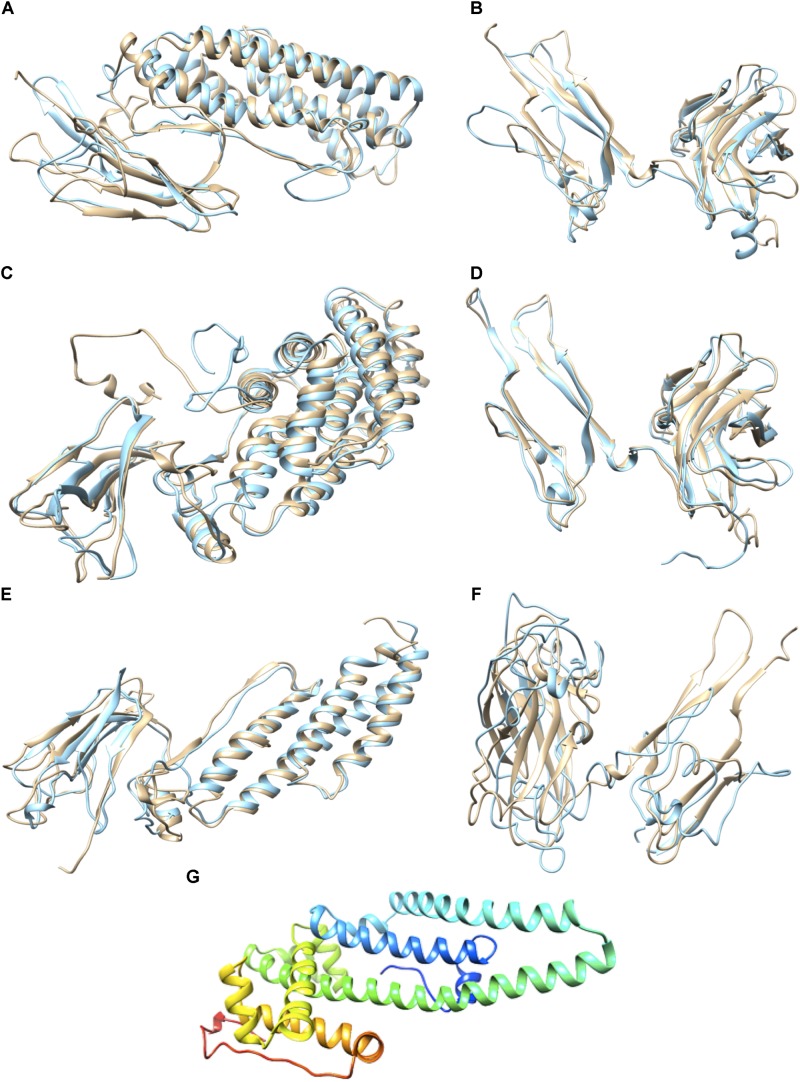
Prediction of the 3D Structures of Cry11Aa and Variants 8, 23, and 79. **(A)** Conserved region of variant 8 in light blue and Cry11Aa in beige, RMSD: 1,084 with 247 aa. **(B)** Non-conserved region of variant 8 in light blue and Cry11Aa in beige. **(C)** Conserved region of variant 23 in light blue and Cry11Aa in beige, RMSD: 1,132 with 488 aa. **(D)** Non-conserved region of variant 23 in light blue and Cry11Aa in beige. **(E)** Conserved region of variant 79 in light blue and Cry11Aa in beige, RMSD: 1,084 with 247 aa. **(F)** Non-conserved region of variant 79 in light blue and Cry11Aa in beige. **(G)** Ribbon representation of the non-conserved region of variant 79 generated using the Robetta server.

The Z-score, ERRAT and Verify3D results obtained for Cry11Aa and variants 8, 23, and 79 are shown in **Supplementary Table 2**. Multiple alignment including structural alignment of Cry11Aa and its variants showed that variants 8 and 23 are similar to their parental protein due to high sequence conservation (**Supplementary Figures 2A,B**). The similarity of these variants with respect to Cry11Aa was 87.4 and 98.9%, respectively. These variants also showed similar structural conformation to Cry11Aa (**Figures 3A–D**). However, variant 79 exhibited structural differences in the non-conserved region compared with its parental protein (**Figures 3E,F**). The similarity of variant 79 with respect to Cry11Aa was 55,7%. The predicted secondary structure in the non-conserved region of variant 79 predominantly contains α helices instead of β-sheets, which are found in the corresponding region of Cry11A. This result was also found based on *ab initio* analysis, thus confirming the high prevalence of α helices in this region (**Figure 3G**).

### The Interactions of Cry11Aa With ALP1 Are Conserved in Variants 8 and 23

The Ramachandran analysis of ALP1 showed that 76% of its residues were in favorable zones according to phi and psi angle positions and were involved in interactions with the parental Cry11Aa protein or its variants. None of the residues of ALP1 positioned in unfavorable zones (**Supplementary Figure 4A**) were involved in interactions with the parental Cry11Aa protein or its variants. The Z-score (-7.27) and the energy obtained from the ProSA-web server were below 0. Both analyses matched the score reported in the PDB crystallographic database, and the interactions were found to be energetically stable (**Supplementary Figures 4B–D**).

**FIGURE 4:**
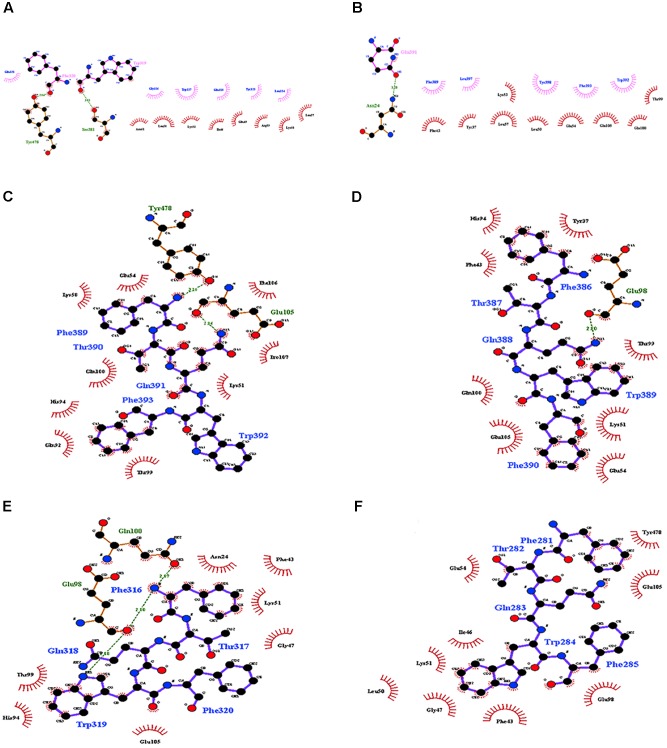
Molecular docking of the interactions of domain II of Cry11Aa and its variants with ALP1. **(A,B)** Interactions formed by 11-amino acid peptides within domain II of Cry11Aa. **(A)** Variant 8 **(B)** Cry11Aa. **(C–F)** Interactions formed by 5-amino acid peptides within domain II of Cry11Aa **(C)** variant 8 **(D)** Cry11Aa **(E)** Variant 23 **(F)** Variant 79.

The interaction of Cry11Aa with ALP1 involves a peptide of 12 amino acids, ^389^FTQWFQSTLYGW^400^, within loop 2 in domain II of Cry11Aa that was conserved in variants 8 and 23 (**Supplementary Figure 2A**). However, in variant 79, the only first five amino acids, ^281^FTQWF^285^, were found (**Supplementary Figure 2B**). The identified interactions of ALP1 with the twelve-amino acid peptide indicated that W^319^ and F^320^ of variant 8 form hydrogen bonds with Y^478^ and S^381^ of ALP1, respectively (**Figure 4A**). The protein complex between variant 8 and ALP1 was also stabilized by five and eight hydrophobic interactions, respectively (**Figure 4A**). For variant 23, seven hydrophobic interactions with eleven amino acids of ALP1 were found (**Supplementary Figure 5**). For Cry11Aa, nine hydrophobic interactions with five amino acids and one hydrogen bond with Q^391^ of ALP1 were found (**Figure 4B**). The identified interactions of ALP1 with the five-amino acid peptide of variant 79 described above were also analyzed for the interactions of ALP1 with Cry11Aa as well as variants 8 and 23. Amino acids F^316^ and W^319^ of variant 8 formed three hydrogen bonds with amino acids E^98^ and Q^100^ of ALP1 (**Figure 4C**) in contrast to the two hydrogen bonds found in the interaction between F^389^ and Q^391^ of the parental Cry11Aa protein with Y^478^ and E^105^ of ALP1 (**Figure 4D**). For variant 23, a single hydrogen bond between G^388^ and Q^98^ of ALP1 was found (**Figure 4E**), whereas variant 79 formed five hydrophobic interactions with nine amino acids of ALP1 (**Figure 4F**).

**FIGURE 5:**
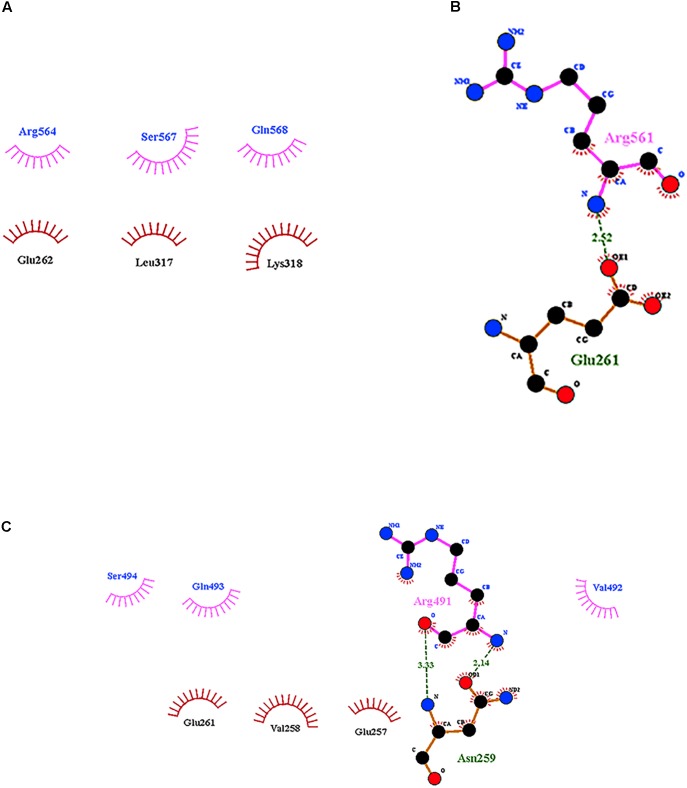
Interactions visualized in LigPlot of ALP1 against peptides of Cry11Aa and its variants 8 and 23. **(A)** Cry11Aa, **(B)** Variant 23, and **(C)** Variant 8.

According to the data for Cry11Aa, the peptide ^564^RVQSQNSGNN^573^, located in the β18β19 region of domain III, was found in all variants with exception of variant 79 (**Supplementary Figure 2C**). The LIGPLOT analysis of Cry11Aa showed three hydrophobic interactions of Cry11Aa with surface-exposed amino acids of ALP1 (**Figure 5A**). However, in variant 23, only one stable interaction through a hydrogen bond between R^561^ of the variant protein and G^261^ of ALP1 was observed (**Figure 5B**). In variant 8, two hydrogen bonds between R^491^ of the variant protein and N^259^ of ALP1 as well as three hydrophobic interactions of R^491^, V^492^, and Q^493^ of the variant protein with V^258^, G^257^, and G ^261^ of ALP1 were formed (**Figure 5C**).

## Discussion

Directed evolution approaches such as phage display, DNA shuffling and staggered extension process shuffling combined with Red/Et homologous recombination have been proposed to increase the activity of *Bt* Cry toxins (Lucena et al., 2014). Other approaches based on phage-assisted continuous evolution (PACE) (Badran et al., 2016), *in vitro* template-change PCR (Shu et al., 2016), site-directed mutagenesis, and error-prone PCR have also been used successfully to identify novel receptors expressed on the surface of insect midgut cells and to understand the effects of different *cry* gene mutations on the mechanism of action of Cry toxins (Lucena et al., 2014). In this study, we report five Cry toxin variants produced via reassembly during DNA shuffling of the *cry11Aa* gene that showed toxic activity against *A. aegypti* and *C. quinquefasciatus* larvae.

DNA shuffling was designed using internal sequences in the parental constructs, including the identification of specific sites for priming during reassembly. We used the upstream *p19* gene to identify the genes that were reassembled from *cry11Aa*. A deletion of 246 bp downstream of the ATG start site was used to recognize those genes reassembled from *cry11Ba*, and an internal sequence corresponding to the specific primers was used to reassemble the variants from the construct containing the *cry11Bb* gene. The *p19* gene was present upstream of the first ATG codon in variants 1, 8, 23, 28, 54, 79, and 81. According to sequence analysis, all variants displayed some degree of identity to *cry11Aa*; this observation indicated that all variants were preferentially reassembled from this parental gene during DNA shuffling (**Table 1**).

Variants 1 and 79 retained toxic activity against *A. aegypti* and *C. quinquefasciatus* despite lacking 8.0 and 11.9 kDa N-terminal regions, respectively. These variants exhibited the highest mutation rates, 15 and 20%, respectively. The mutations found in variant 1 were not in regions involved in pore formation or toxin-receptor interactions, explaining the toxicity of variant 1 to *A. aegypti* based on bioassays. The insertion of 22 amino acids at the end of the C-terminus with seven substitutions did not affect the toxic activity of variant 1. The results of the bioassays with *C. quinquefasciatus* larvae showed that variant 1 has similar toxic activity to Cry11Aa but 7.63-fold lower toxic activity than Cry11Bb. In the other hand, toxic activity of variant 79 was unexpected, despite its numerous mutations, its toxic activity against *A. aegypti* and *C. quinquefasciatus* was high (**Figure 2**). The deduced amino acid sequence of variant 79 did not show mutations in helices α-4 and α-5, which are implicated in pore formation, or in regions involved in toxin-receptor interactions, such as loop α8 and strand β4 (**Supplementary Figure 2C**). However, among the 260 amino acids at the C- terminus that were modified in variant 79, we found five amino acids located in loop α-2 (F^281^, T^282^, Q^283^, W^284^, and F^285^) that generate nine hydrophobic interactions with ALP1 (**Figure 4F**). These five amino acids in Cry11Aa and in variants 8 and 23 also form more stable interactions with ALP1 than hydrogen bonds (**Figures 4C–E**). Additionally, according to 3D and secondary structural analyses, the non-conserved C-terminal region of variant 79 has an unusual α-helix conformation (**Figure 3G**). The structural conformation of domains II and III of variant 79 has not previously been observed in Cry toxins; according to BLASTP analysis, this protein region is completely new. Therefore, we cannot discard the possibility that interactions of loop α8 in domain I of variant 79 with loop α-2 of ALP1 could be sufficient to explain the toxicity of this variant.

Variants 23 and 81 contain N-terminal deletions of 0.33 and 5.5 kDa, respectively. The mutation rates of variants 23 and 81 were less than those of other variants, and these two variants displayed 6.8- and 11.6-fold lower toxic activity against *A. aegypti* larvae, respectively, relative to Cry11Aa (**Table 1** and **Figure 2**). However, these variants retained high activity against *C. quinquefasciatus*. The LC_50_s of variants 23 and 81 for *C. quinquefasciatus* are similar with the values that have been reported for Cry11Aa (van Frankenhuyzen, 2009) and were comparable to those of the parental protein in our bioassays. Loop α8, strand β4 and loop 3 in domain II of these variants did not contain mutations, but differences in docking analysis results for variant 23 compared to the other variants could explain the moderate toxicity of variant 23 to *A. aegypti*. In loop 2, mutation S^392^F caused loss of the hydrogen bond formed via the interaction between Q^391^ of the wild type protein and N^24^ of ALP1, potentially reducing energetic stability and decreasing the specificity of the intermolecular interactions. In the same region, in variant 23, four out of the nine amino acids of ALP1 that initially interacted with the wild type Cry11Aa toxin were involved in hydrophobic interactions; this evidence suggests that these changes could affect the stability of the toxin-receptor interaction (**Supplementary Figure 5**). The interaction between the β18-β19 region in domain III of variant 23 and ALP1 also appears to be characterized by a lack of hydrophobic interactions and the formation of a stable hydrogen bond between R^561^ of variant 23 and Q^261^ of ALP1 (**Figure 5B**). The observed differences in the interaction of ALP1 with wild type Cry11Aa and variant 23 based on docking analysis could be explained by the peptide conformations in the original model (Cry11Aa and variant 23 were considered as rigid peptides folded in the original conformation). In this manner, a technological limitation of docking analysis was overcome by producing interactions with the complete protein. Based on these findings, we suggest that the changes found in loop 2 of domain II and the interactions observed in the β18-β19 region of domain III affect the stability of the interaction of variant 23 with ALP1, thereby producing the moderate toxicity of variant 23 to *A. aegypti*.

Variant 8 was the most important of this study despite a deletion of 8.0-kDa at the N-terminus and similar 3D structure to Cry11Aa (**Figures 3A,B**), it showed an increased toxic activity of 6.09 times compared to Cry11Aa toward *A. aegypti* without significant differences against *C. quinquefasciatus* (**Figure 2**). Importantly, this variant did not show substitutions in regions involved in pore formation such as helices α4 and α5 neither in regions involved in toxin-receptor interaction such as loop α8 and strand β4. However, three substitutions T^453^A, R^456^G, and P^462^R in loop 3 located in domain II were found (**Supplementary Figure 2A**). In Cry11Aa, this loop did not shown any interaction using the synthetic peptide ^447^LTYNRIEYDSPTTEN^461^ in binding assays in the presence of *A. aegypti* BBMV (Fernandez et al., 2009). So far, mutations in loop 3 have been reported in Cry4Ba toxin that produces an increase of toxicity of 1.38 and 700 times toward *A. aegypti* and *C. quinquefasciatus*, respectively (Abdullah et al., 2003). In our study, the interactions found by docking analysis in loop 2 of domain II and strands β18-β19 in domain III suggest a role in the stability of the interaction with ALP1. The formation of two hydrogen bonds in loop 2 of domain II produced by W^319^ and F^320^ with two amino acids of ALP1 including one hydrophobic interaction (Q^321^) in the same cavity (**Figure 4A**), as well as two hydrogen bonds formed in domain III by R^491^ with N^259^ of ALP1 and three hydrophobic interactions, could explain the toxicity toward *A. aegypti* mediated by ALP1 (**Figure 4C**).

The N-terminal deletions found in variants 1, 8, 23, 79, and 81 were between 3 and 108 amino acids in length (0.33 and 11.8 kDa). However, these deletions are not implicated in the differences in toxic activity against *A. aegypti* and *C. quinquefasciatus* larvae between variants. Only one study has reported an N-terminal deletion in the Cry11Aa toxin where a truncated protein lacking 9.6 kDa was non-toxic to *A. aegypti* (Pang et al., 1992). In other Cry toxins such as Cry2a, deletion of 42 amino acids at the N-terminus increased the toxic activity against *Spodoptera littoralis*, *Helicoverpa armigera*, and *Agrotis ipsilon* (Mandal et al., 2007). Furthermore, in Cry1Ac, a deletion of 56 amino acids at the N-terminus, which included helix α-1, increase the toxic activity against *Pectinophora gossypiella* by 107-fold (Mandal et al., 2007) and against *Plutella xylostella* and *Ostrinia nubilalis* by 350-fold (Tabashnik et al., 2011). However, the toxic activity of Cry4Ba against *A. aegypti* was abolished when more than 38 amino acids were removed from the N-terminus (Pao-intara et al., 1988) and, in the case of chimeric proteins formed by a fusion of N-terminus of Cry4Ba and the C-terminus of Cry1Ac, an increase of toxicity against *C. pipiens* larvae was observed (Zghal et al., 2017). Although these findings confirm the importance of the N-terminal region in the toxicity of Cry proteins, the N-terminal deletions found in our variants did not affect their toxic activity against *A. aegypti* or *C. quinquefasciatus* larvae.

The difference in toxicity against *A. aegypti* and *C. quinquefasciatus* (**Figure 2**) could be explained by the presence of compounds either in midgut juice or membranes-bond proteases. Although the roles of these compounds and proteases have not been tested in variants obtained by DNA shuffling, there is evidence that the capacity to processing the protoxin depends on specific proteases located in the larval midgut and favored by alkaline conditions (Ben-Dov, 2014). In Cry11Aa toxin, the treatment with proteases generates fragments with different molecular weight and toxic activity against *A. aegypti* (Revina et al., 2004; de Barros Moreira Beltrao and Silva-Filha, 2007), whereas in *C. quinquefasciatus*, the processing pattern differ from those that are active to *A. aegypti* (Dai and Gill, 1993). Therefore, we suggest that the toxicity differences found in *A. aegypti* and *C. quinquefasciatus* could be explained by mechanism that dependent on the host.

Overall, the data presented in this report indicate that the N-terminal deletions observed in all variants did not affect their toxicity to *A. aegypti* or *C. quinquefasciatus*. Variant 8, which contained several substitutions in domains II and III, was the most interesting variant produced in this study due to its high toxicity to the two mosquito species. These findings confirm the importance of these domains in Cry toxin-receptor interactions and in Cry protein toxicity. The substitutions found in variants 8 and 23 provide new information about the role of loops 2 and 3 of domain II in Cry toxin-ALP1 interactions. Importantly, the α helix conformation of the C-terminus of variant 79 based on secondary structure analysis corresponds to a new protein structure with toxic activity. We believe that DNA reassembly via DNA shuffling following random fragmentation could be a good strategy to generate random mutations in specific *cry* genes to design new and more potent toxins.

## Author Contributions

AF conceived the study, was in charge of overall direction and planning and wrote the manuscript with input from all authors. MS-B carried out the experiments and worked out almost all of the technical details. GM performed directed evolution techniques and sequencing analysis. KR assisted with MS-B measurements and bioassays. SO contributed to electronic microscopy analysis and devised the project. RO carried out the molecular docking analysis. DG contributed to the analysis of 3D structure prediction and validation and secondary structure analysis of non-conserved regions. CM contributed to the interpretation of the 3D structure prediction and docking results.

## Conflict of Interest Statement

The authors declare that the research was conducted in the absence of any commercial or financial relationships that could be construed as a potential conflict of interest.

## Footnotes

1 http://blast.ncbi.nlm.nih.gov/Blast.cgi

2 http://www.expasy.org

3 https://www.r-project.org/

4 http://zhanglab.ccmb.med.umich.edu/I-TASSER/

5 http://swissmodel.expasy.org/workspace/?func=tools_structureassessment1

6 https://prosa.services.came.sbg.ac.at/prosa.php

7 http://services.mbi.ucla.edu/ERRAT/

8 http://services.mbi.ucla.edu/Verify_3D/

9 http://www.compbio.dundee.ac.uk/jpred/

10 http://robetta.bakerlab.org/

11 http://autodock.scripps.edu/resources/adt

12 http://vina.scripps.edu/

13 http://www.ebi.ac.uk/thornton-srv/software/LIGPLOT/
