# Supplementary material for: Toxic Activity, Molecular Modeling and Docking Simulations of Bacillus thuringiensis Cry11 Toxin Variants Obtained via DNA Shuffling
Source: Front Microbiol. 2018 Oct 17;9:2461. doi: 10.3389/fmicb.2018.02461 (PMC6199390; doi:10.3389/fmicb.2018.02461)
Supplement: Supplementary file 1 [file Data_Sheet_1.docx]

Supplementary Material

Toxic activity, molecular modeling and docking simulations of *Bacillus thuringiensis* Cry11 toxin variants obtained via

DNA shuffling

**Alvaro Mauricio Florez ^1,^*, Miguel O. Suarez-Barrera ^2,3^, Gloria M. Morales ^2^, Karen Viviana Rivera^2^, Sergio Orduz^4^, Rodrigo Ochoa^5^, Diego Guerra^5^, Carlos Muskus^5^**

*** Correspondence:**Alvaro Mauricio Florez
amflorez@microbiomas.org

# Supplementary Figures and Tables

**(A)**

**(B) (C) (D) (E)**

1 2 3 4 5 6 7 8 9 10 11 12 13 14 15

250

500

750

1000

1500

2000

2500

3000

3500

4000

25

50

75

100

125

250

175

150

300

400

500

2000

1500

1000

750

2000

1500

1000

750

200

kb

kb

kb

kb


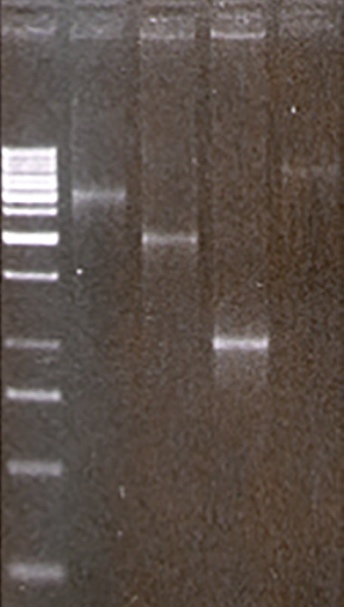

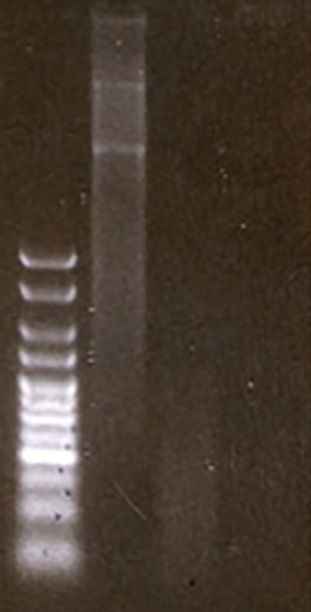

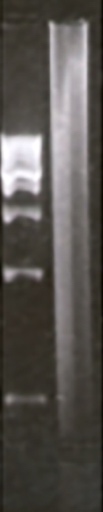

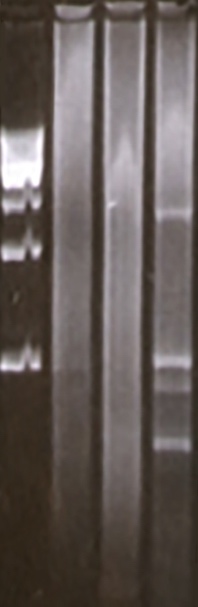


**Supplementary Figure 1. Amplification, DNase I Digestion, Assembly and Reassembly of *cry11* Genes.** **(A)** Image of 0.8% agarose gel electrophoresis of PCR products obtained with PCR4 primers for *cry11Aa*, *cry11Ba*1 and *cry11Ba*2 and pGE7 primers for *cry11Bb*. Lane 1 and 5: Molecular weight 1 kb (Fermentas), Lane 2: *cry11Aa*, Lane 3: *cry11Ba*-2, Lane 4: *cry11Ba*-1, Lane 6: *cry11Bb*. **(B)** DNA shuffling for *cry11* genes. Lanes 1, 10 and 12: Molecular weight 1 kb (Fermentas)**.** Purified PCR products obtained with PCR4 primers for *cry11Aa*, *Ba*1 and *Ba*2 and pGE7 primers for *cry11Bb*, Lane 2: *cry11Aa*, Lane 3: *cry11Ba1*, Lane 4: *cry11Ba2*, Lane 5: *cry11Bb*. **(C)**. Image of 2.5% agarose gel electrophoresis of the products of DNase I (0.0006 U) digestion. Lane 6: Molecular weight 25 bp (Bioline), Lane 7: digestion products after 7 min, Lane 8: digestion products after 8 min, Lane 9: digestion products after 9 min. **(D).** Image of 0.8% agarose gel electrophoresis. Lane 11: assembly of the DNase I-treated products from lanes 8 and 9 performed without primers. **(E).** Image of 0.8% agarose gel electrophoresis of the products of reassembly with primers PCR4 and PGE7. Lane 13: products of reassembly with primers PCR4F/R, Lane 14: products of reassembly with primers PGE7F/R, Lane 15: products of reassembly with primers PCR4F and PGE7R.

**
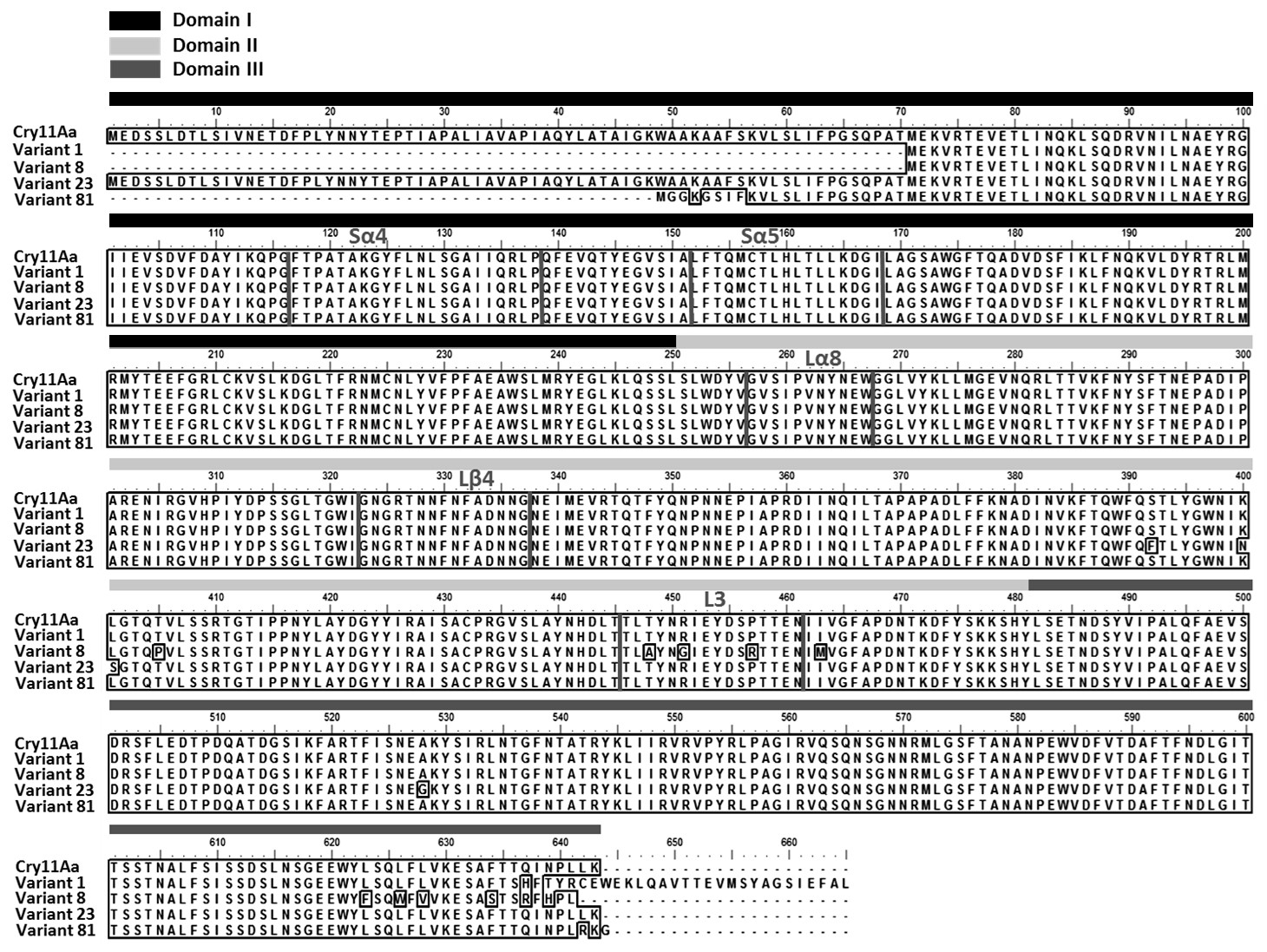
**

**(A)**


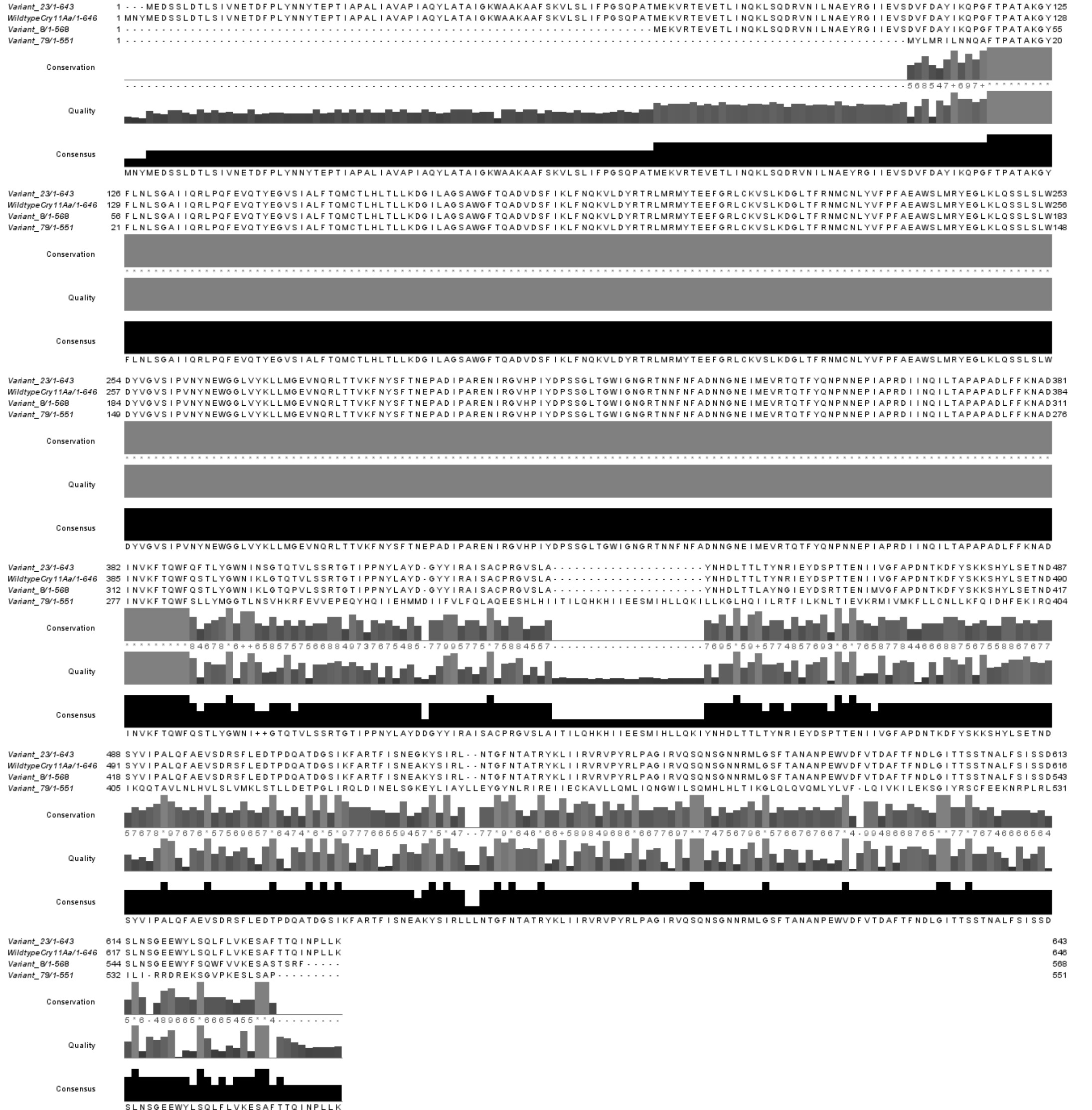


**(B)**


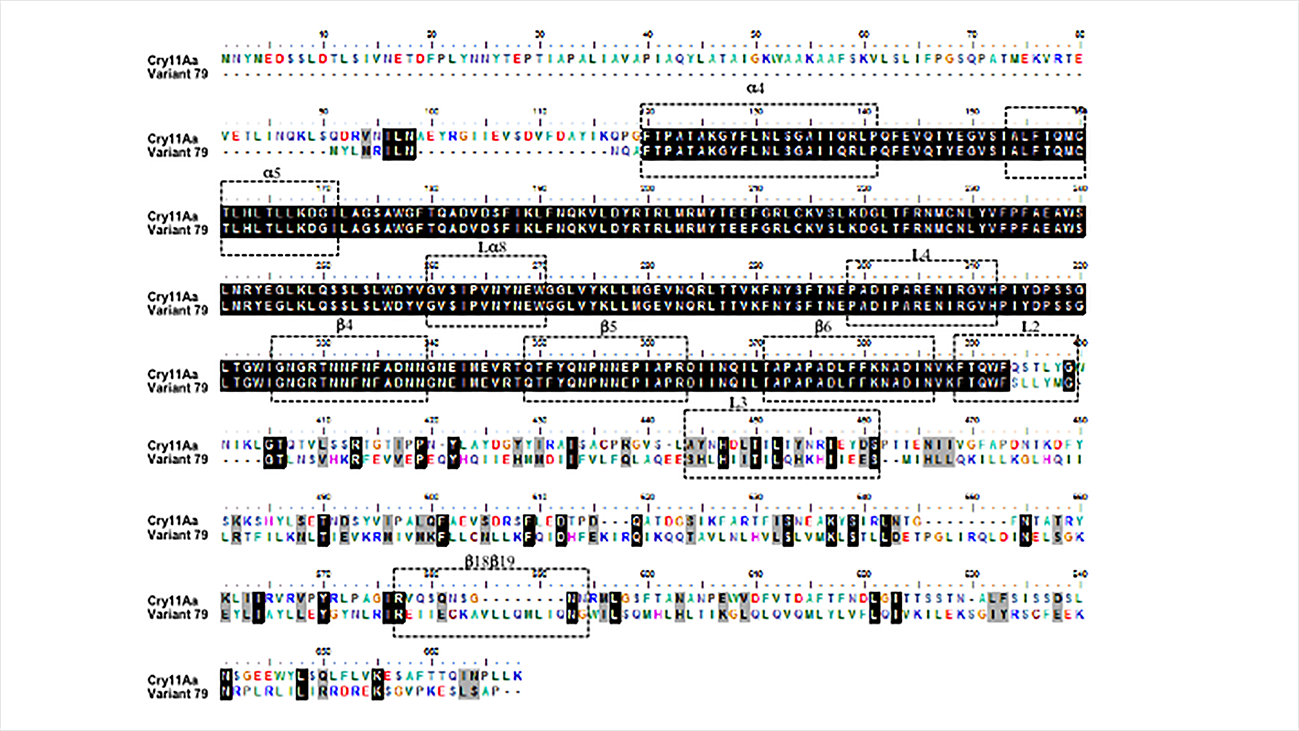


**(C)**

**Supplementary Figure 2. Alignment of Deduced Amino Acid Sequences of Variants 1, 8, 23, 79 and 81. (A)** Variants 1, 8, 23, and 81. The domains are defined above the amino acid sequence in horizontal gray bars. Helices α4 and α5 are denoted as α4 and α5, respectively. Loop α8, strand β4 and loop 3 are denoted as Lα8, β4 and L3, respectively. Substitutions are indicated by closed boxes. **(B)** The conservation, quality and consensus sequence similarity of variants 8, 23, and 79. **(C)** Alignment of the deduced amino acid sequences of wild type Cry11Aa and variant 79. Helix regions are denoted as α4 and α5. Loop regions are denoted above the deduced amino acid sequence in dotted boxes as Lα8, L1, β4, β5, β6, L2, L3 and β18-β19. Identical amino acids are shown in white color.

**kDa**

**MW 1 2 3 4 5 6 7 8**

**
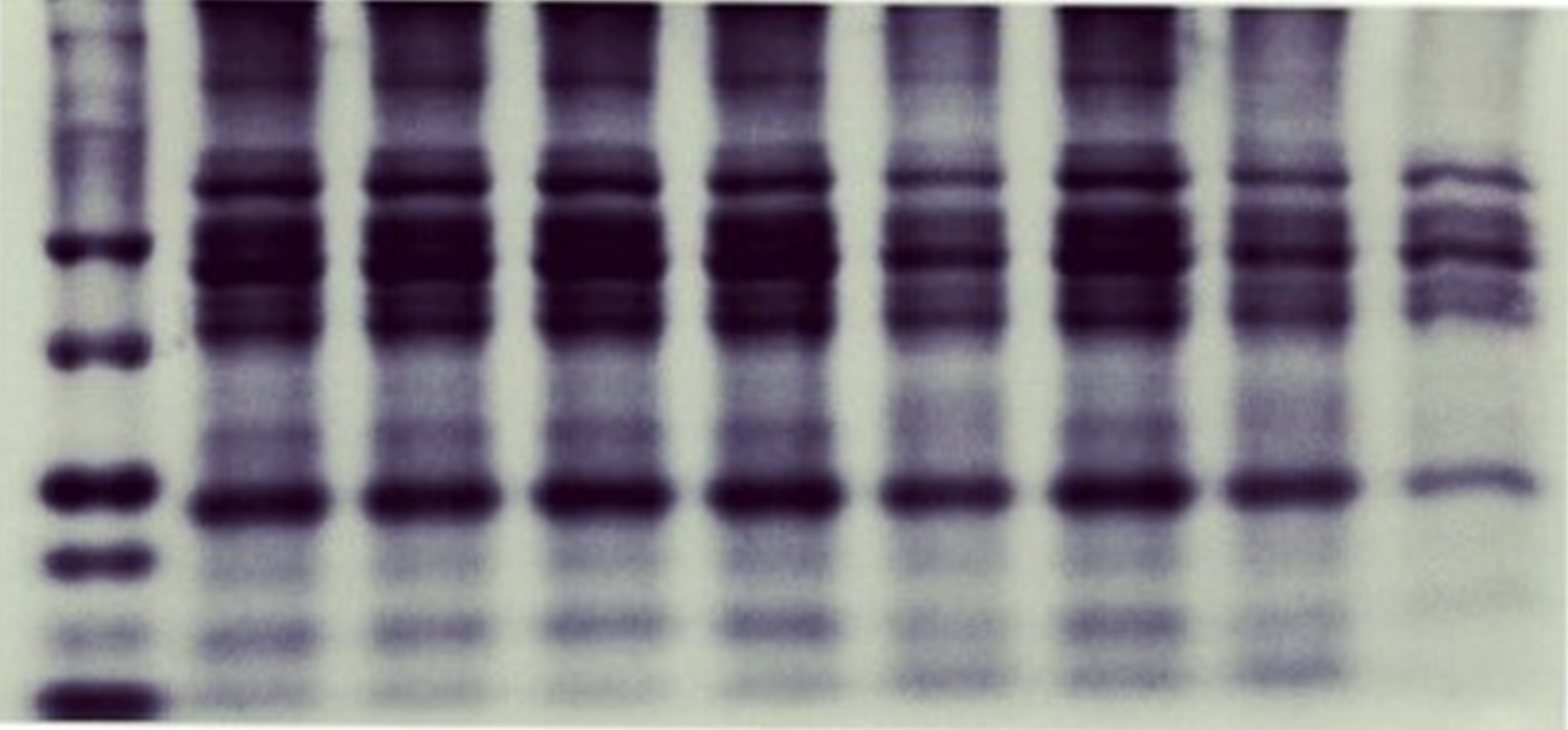
**

50

100

75

**(A)**

**Cry11Aa**


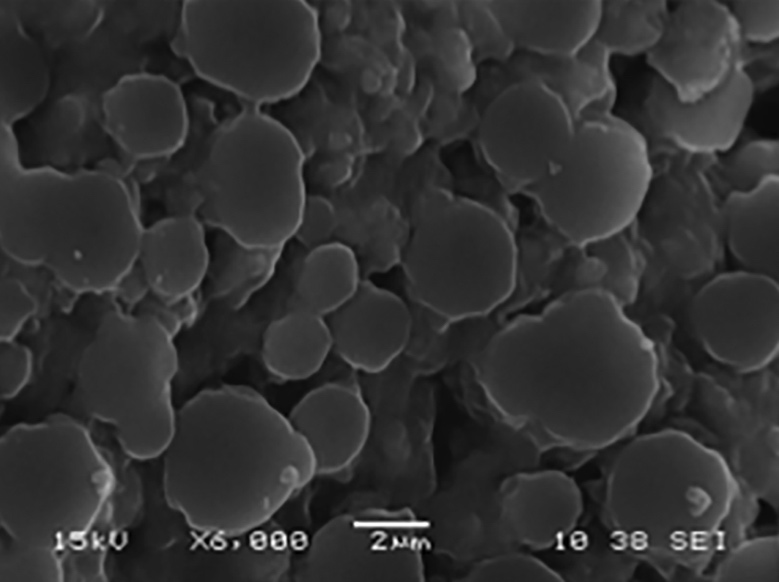


**Variant 1**

**Cry11Bb**

**Variant 23**

**Variant 8**

**Variant 79**

**Variant 81**

**BMB171**


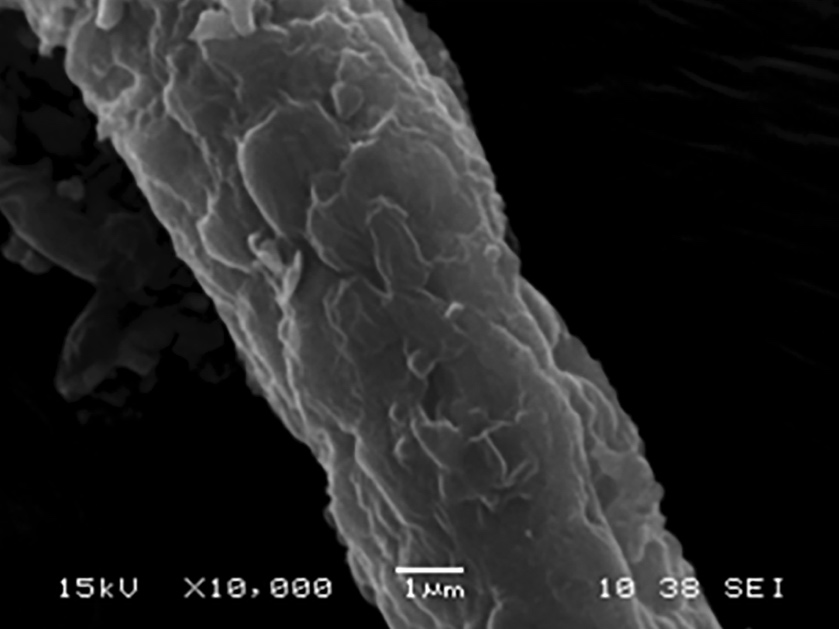


**C**


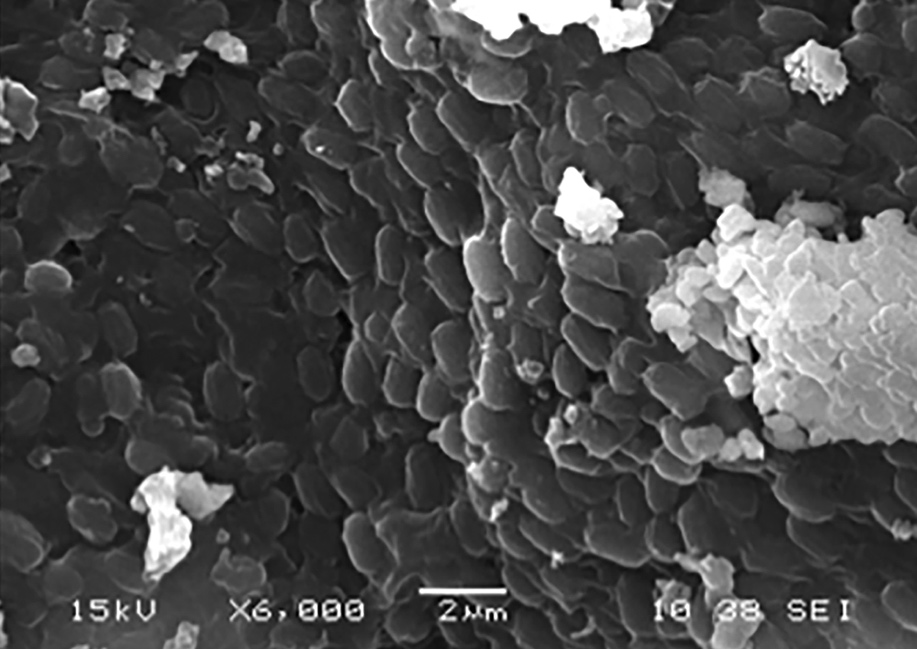


**C**


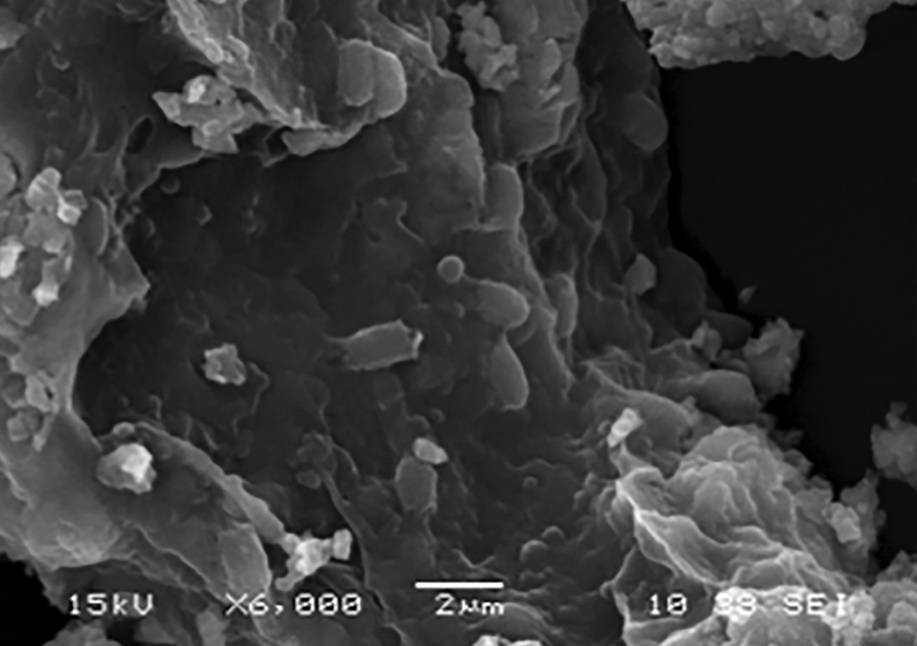


**C**


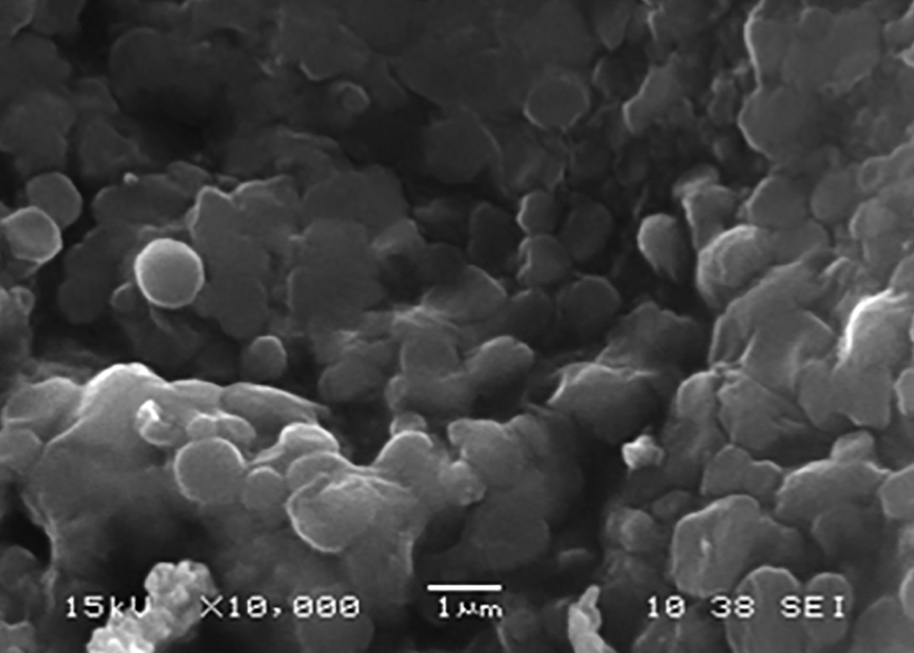


**C**


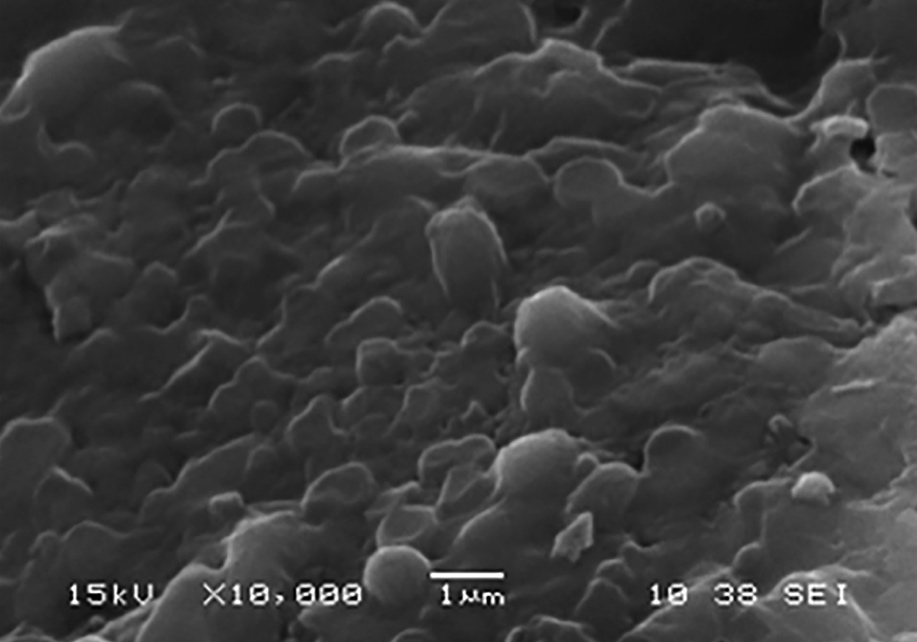


**C**


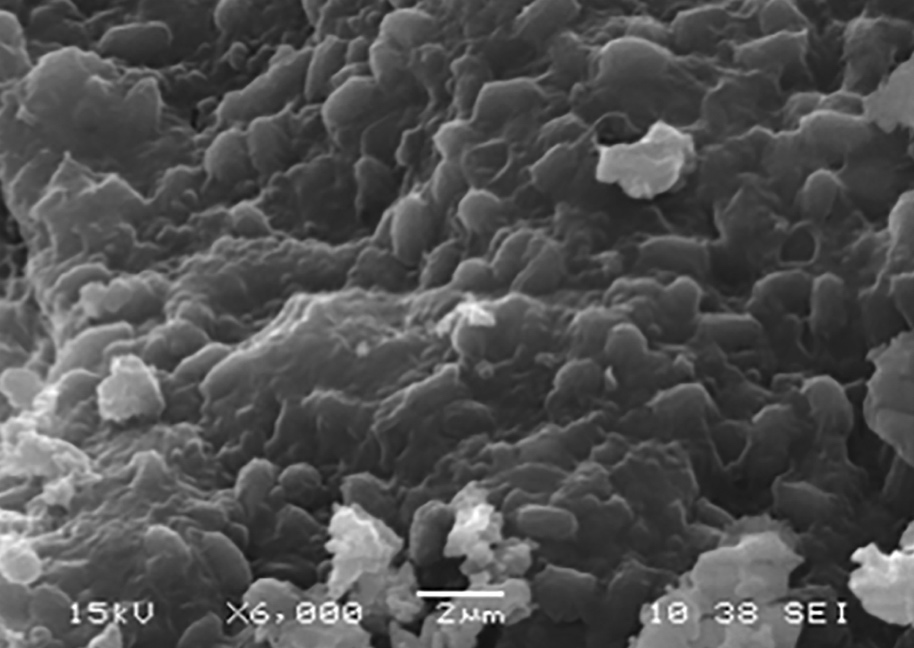


**C**


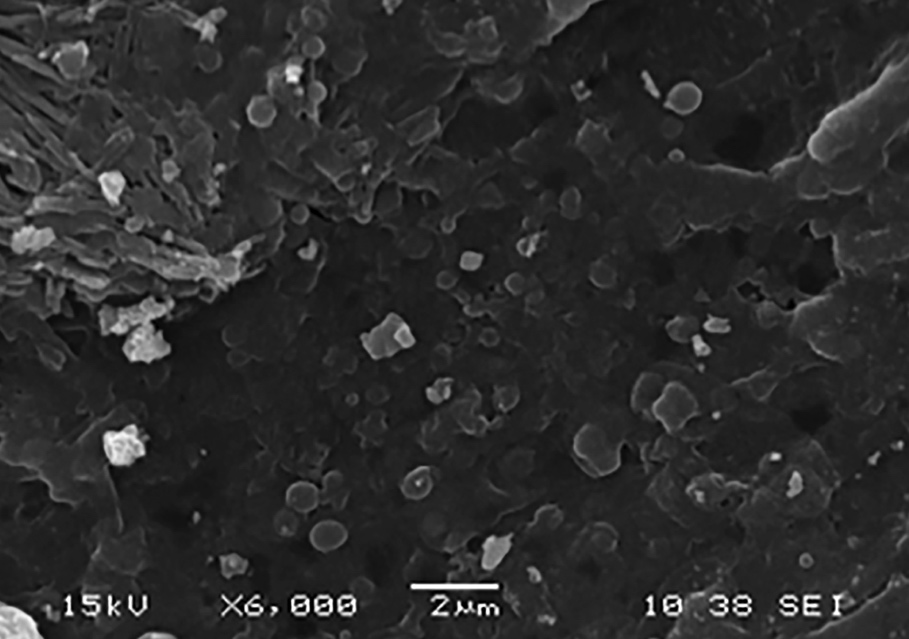


**C**

**(B)**

**(B)**

**Supplementary Figure 3. Protein Composition of Crystals Produced by Variants 1, 8, 23, 79 and 81.** **(A)** SDS-PAGE analysis. Lane 1. Kaleidoscope molecular weight standards (BioRad^TM^); Lane 2: variant 1; Lane 3: variant 8; Lane 4; variant 23; Lane 5: variant 79; Lane 6: variant 81; Lane 7: Cry11Aa; Lane 8: BMB171. **(B)** SEM analysis. Photomicrographs of variants 1, 8, 23, 79 and 81. Controls are Cry11Aa, Cry11Bb and the acrystaliferous strain BMB171. Scale bars: 1 and 2 μm. White arrows and the letter C indicate crystals.


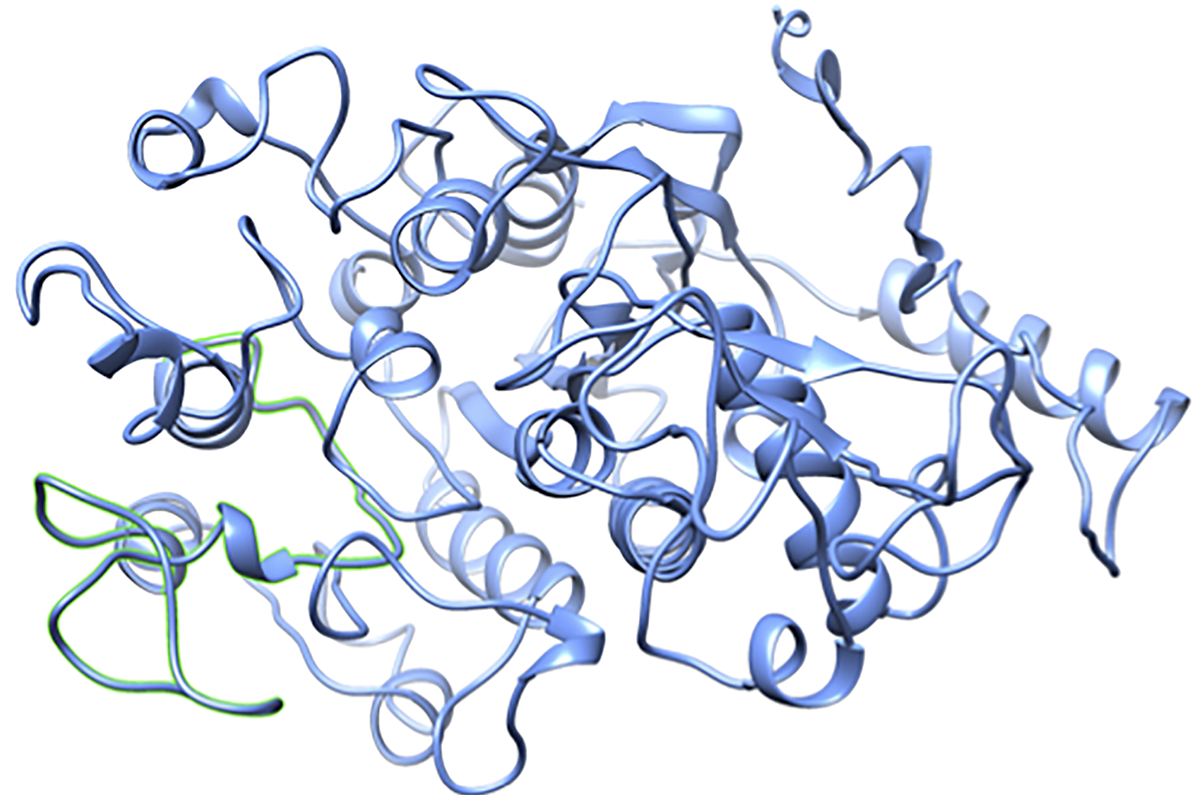

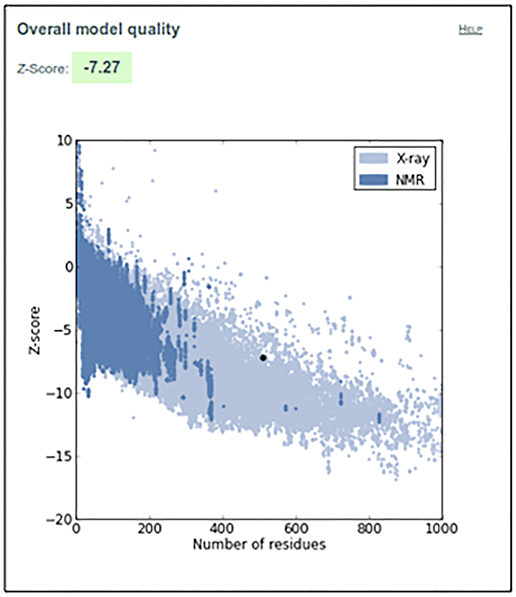

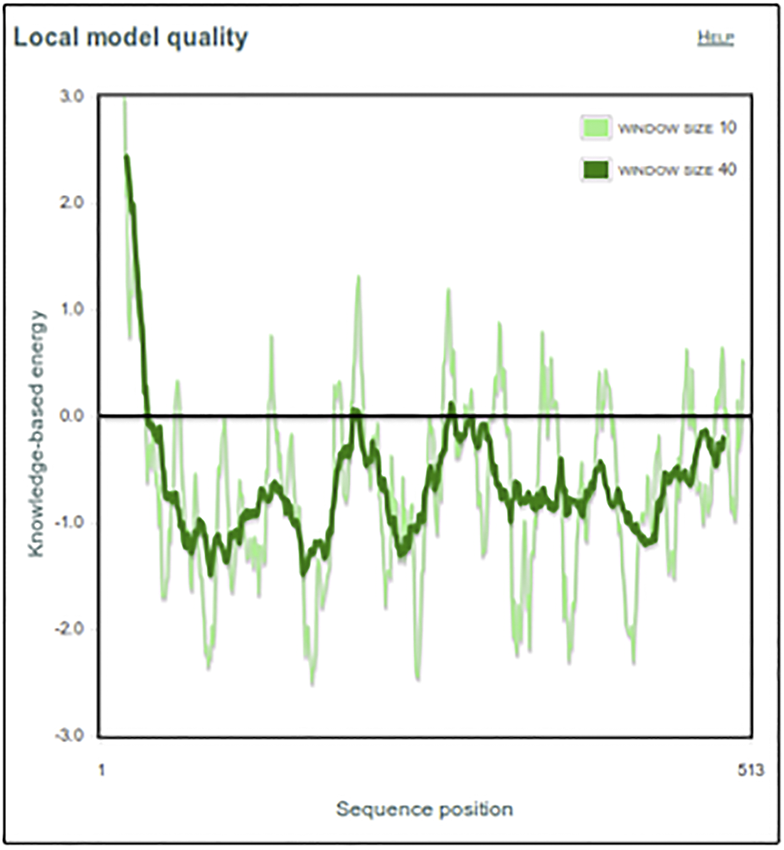

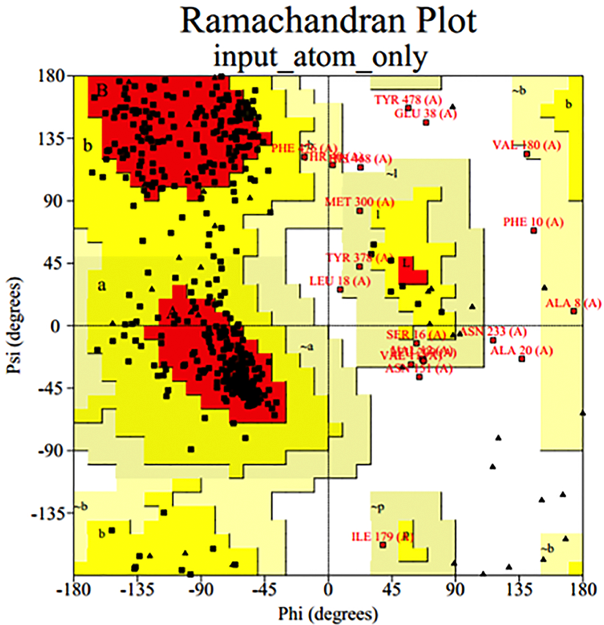


**(A)**

**(B)**

**(C) (D)**

**Supplementary Figure 4. Prediction of the 3D Structure of ALP1. (A)** Ramachandran plot analysis generated using the program Procheck on the SwissModel Server. **(B)** Energy curve. **(C)** Predicted structure of ALP1. The flexible region that was selected for interaction with Cry11Aa is shown in green. **(D)** Z–score analysis.


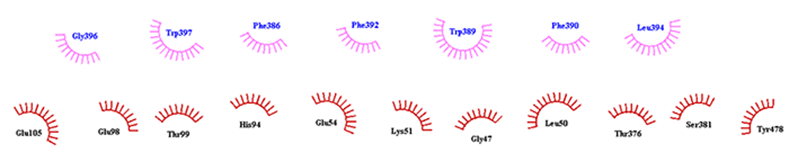


**Supplementary Figure 5. Molecular Docking of the Interactions of Domain II variant 23 with ALP1.** Interactions formed by 12-amino acid peptides within domain II of Variant 23.

**Supplementary Table 1. List of Strains, Constructs and Primers used to Perform DNA Shuffling**

| Strain | Construct name | Gene | Primers | bp |
| --- | --- | --- | --- | --- |
| *E. coli* DH5αTOP10^©^ (Life Technologies) | pTOAa | *cry11Aa* | PCR4F: 5´-ATAACAATTTCACACAGGA-3´  PCR4R: 5´-TTGTAAAACGACGGCCAGTG-3´ | 2,516 |
|  | pTOBa-1 | *cry11Ba* |  | 1,600 |
|  | pTOBa-2 | *cry11Ba* |  | 780 |
|  | PTOBb | *cry11Bb* |  | 3,700 |
| *E. coli* JM109® (Promega) | pGEBb | *cry11Bb* | pGE7F: 5´-GATGTGCTGCAAGGCGATT-3´  pGE7R: 5´-TTACGCCAAGCTATTTAGGTG-3´ | 3,500 |

**Supplementary Table 2. Scores for Cry11Aa and Variants Obtained from Analysis Servers.**

| Protein | Z-score | ERRAT | Verify3D |
| --- | --- | --- | --- |
| Cry11Aa | -6,49 | 51,334 | 71,05 |
| Variant 8 | -5,72 | 51,075 | 75,18 |
| Variant 23 | -6,71 | 59,49 | 80,56 |
| Variant 79 | -5,28 | 43,068 | 59,99 |
